# Tilianin: pharmacological potential, mechanisms of action, and future perspectives in traditional and modern medicine

**DOI:** 10.1186/s13020-025-01245-4

**Published:** 2025-11-19

**Authors:** Mohammed A. Abdel-Rasol, Ruwaidah A. R. Abbas, Wael M. El-Sayed

**Affiliations:** 1 Department of Zoology, Faculty of Science, Ain Shams University, Abbassia, Cairo, 11566 Egypt; 2 Department of Pathological Analysis, College of Applied Science, University of Fallujah, Fallujah, Iraq

**Keywords:** Anticancer, Antioxidant, Flavonoids, Metabolic disorder, Neuroprotection, Pharmacological properties

## Abstract

**Purpose:**

This review evaluates the therapeutic potential of tilianin, a flavonoid glycoside from medicinal plants, in managing chronic diseases such as cancer, cardiovascular diseases, neurodegenerative disorders, and metabolic syndromes, while addressing key research gaps for clinical application.

**Methods:**

A comprehensive review of literature was conducted using PubMed, focusing on studies published between 2000 and 2025. Peer-reviewed articles examining tilianin's pharmacological properties, molecular mechanisms, and clinical applications were selected, with an emphasis on bioavailability, pharmacokinetics, and combination therapies.

**Results:**

Tilianin demonstrated a broad range of pharmacological effects, including antioxidant, anti-inflammatory, neuroprotective, cardioprotective, and anticancer activities. It showed promise in treating neurodegenerative diseases, mitigating ischemic injury, and regulating glucose and lipid metabolism. Additionally, tilianin exhibited hepatoprotective and renoprotective effects in animal models of non-alcoholic fatty liver disease and chronic kidney disease. However, challenges such as poor bioavailability, limited clinical trials, and the need for optimized drug delivery systems persist.

**Conclusion:**

The novelty of this review lies in its holistic approach, consolidating evidence from in vitro, animal model, and ethnopharmacological studies, while addressing the need for clinical trials and improved pharmacokinetics. This review expands the scope to include tilianin’s effects on neurodegenerative diseases, cancer, and metabolic syndrome. While tilianin shows promising therapeutic potential, its clinical application is limited by bioavailability issues. Future research should focus on optimizing pharmacokinetics, advancing drug delivery systems, and conducting well-designed clinical trials.

## Methodology

This narrative review adopted a structured, though not systematic, approach to identifying relevant literature. To capture a comprehensive overview of tilianin research, studies published between 2000 and 2025 were searched primarily in the PubMed database. Search terms included “tilianin,” “flavonoid glycosides,” “neuroprotective effects of tilianin,” “tilianin in cancer,” and “bioavailability,” combined with Boolean operators to refine results. Reference lists of relevant papers were also screened manually to identify additional studies.

For study selection, only peer-reviewed articles in English were considered. Eligible studies included experimental, preclinical, and clinical research, as well as systematic reviews and meta-analyses that discussed tilianin’s pharmacological properties, mechanisms of action, pharmacokinetics, bioavailability, and therapeutic applications. Studies were excluded if they were unrelated to tilianin’s pharmacological effects, lacked experimental evidence, were review/commentary pieces without new data, or were published outside the specified timeframe.

In total, 101 original research studies were identified. Of these, 90 English-language studies were included. Eleven non-English studies were excluded: nine focused mainly on the separation and identification of tilianin from plants, and two Chinese-language studies (PMID: 23847962 and 22803383), though relevant, were excluded due to language limitations. While exclusion of non-English studies may introduce language bias, the impact on overall conclusions was considered minimal.

As a narrative review, this work provides a broad, integrative synthesis of tilianin’s pharmacological properties and therapeutic potential. Potential limitations include publication bias (underreporting of negative or null findings) and heterogeneity across included studies (e.g., disease models, dosages, formulations).

## Introduction

Natural products have long been a cornerstone of both traditional and modern medicine, offering a rich variety of bioactive compounds with significant therapeutic potential. Derived primarily from plants, fungi, and other organisms, these compounds have been used for millennia to treat various ailments, laying the groundwork for many pharmacological advancements [1]. Among these, flavonoids stand out as a diverse group of plant-derived secondary metabolites, celebrated for their wide-ranging health benefits. Beyond their role in plant defense against pathogens, oxidative stress, and UV radiation, flavonoids exhibit potent antioxidant, anti-inflammatory, and disease-modulating properties, making them a focal point of contemporary pharmacological research [2]. Found abundantly in fruits, vegetables, tea, and medicinal plants, flavonoids are widely recognized for their ability to combat oxidative stress, reduce inflammation, and alleviate degenerative disorders. Their complex molecular structures and versatile pharmacological effects make them invaluable in drug discovery, offering therapeutic potential that extends far beyond their antioxidant and anti-inflammatory activities. By influencing numerous cellular pathways, flavonoids address complex diseases where conventional treatments often fall short [3].

Tilianin, a flavonoid derived from *Agastache rugosa,* *Agastache mexicana*, and *Dracocephalum moldavica,* exemplifies this dual legacy of traditional and modern medicine. It has notable pharmacological properties and a long history of use in traditional practices, particularly within Traditional Chinese Medicine (TCM) and Mexican herbalism. In TCM, *Agastache rugosa* (Japanese mint) has been valued for its cooling and anti-inflammatory effects, traditionally used to treat respiratory conditions such as coughs, colds, and asthma, as well as digestive disturbances like bloating and indigestion. Similarly, *Dracocephalum moldavica* (Moldavian balm) is recognized for its calming and anti-inflammatory actions, frequently employed to reduce anxiety, stress, and insomnia, while also supporting digestive health. In Mexican herbalism, *Agastache mexicana* has been widely used to alleviate gastrointestinal disorders and to promote relaxation and mental clarity. The therapeutic value of these plants is closely linked to their tilianin content, which contributes significantly to their antioxidant, anti-inflammatory, and neuroprotective properties. Ethnobotanical studies further support the use of *Agastache* species and *Dracocephalum moldavica* in mitigating oxidative stress, inflammation, and neurodegenerative conditions, highlighting tilianin’s longstanding role in traditional medicine [4].

Modern pharmacological research has expanded this foundation, elucidating tilianin’s molecular structure and mechanisms of action. Its glycosylated form enhances solubility, bioavailability, and stability, enabling it to interact with diverse molecular targets and modulate key processes such as oxidative stress, inflammation, immune responses, and metabolic regulation [5]. The presence of a glycosidic bond further supports its pharmacokinetic profile, contributing to sustained activity across multiple systems [6].

One of tilianin’s most notable attributes is its potent antioxidant activity. It effectively scavenges reactive oxygen species (ROS), which are implicated in aging, cancer, cardiovascular diseases, and neurodegeneration, while simultaneously boosting endogenous antioxidant defenses such as heme oxygenase-1 (HO-1) [7]. This dual mechanism provides both immediate and long-term cellular protection, positioning tilianin as a candidate for treating diseases driven by oxidative damage [8]. In addition to its antioxidant effects, tilianin exhibits strong anti-inflammatory properties. By suppressing pro-inflammatory cytokines such as tumor necrosis factor-alpha (TNF-α) and interleukins, and inhibiting nuclear factor-kappa B (NF-κB) signaling, tilianin helps mitigate chronic inflammation—a hallmark of autoimmune, cardiovascular, and metabolic diseases [9, 10]. Its immunomodulatory effects further highlight its potential in managing inflammatory conditions [11].

Tilianin’s cardioprotective effects are also noteworthy, particularly in protecting against ischemic injury. It regulates mitochondrial function, reduces oxidative damage, and influences apoptosis pathways, offering protective benefits for heart tissue and promoting recovery. Studies in animal models have demonstrated tilianin’s ability to modulate proteins like Bax and Bcl-2, which are crucial for maintaining cellular integrity, reinforcing its potential in cardiovascular therapy [12].

neuroprotective potential of tilianin adds to its growing therapeutic appeal. It enhances gamma-aminobutyric acid (GABA) neurotransmission, shields neurons from oxidative stress, and reduces neuroinflammation—critical factors in neurodegenerative diseases like Alzheimer’s and Parkinson’s [13]. By reducing excitotoxicity and neurotoxic protein aggregation (e.g., beta-amyloid), tilianin could help prevent cognitive decline. In the realm of cancer therapy, tilianin has shown promise by inhibiting tumor growth through modulation of key signaling pathways, including PI3K/Akt and MAPK. It induces apoptosis and suppresses cell proliferation, offering a natural and less toxic alternative to conventional cancer treatments [14].

Tilianin’s benefits extend to metabolic health as well. It regulates glucose and lipid metabolism, improves insulin sensitivity, and helps control blood glucose levels, making it a promising candidate for managing diabetes and metabolic syndrome [15]. Its hepatoprotective effects further enhance its therapeutic potential, alleviating liver damage caused by oxidative stress and inflammation, and improving liver function in conditions like metabolic dysfunction-associated non-alcoholic fatty liver disease (MAFLD) and alcohol-induced liver damage [16]. Additionally, tilianin holds promise in mental health, where it has been shown to improve cognitive function, reduce anxiety, and enhance mood by modulating neuroinflammatory processes and GABAergic pathways [13, 17].

Despite this broad therapeutic promise, important challenges remain. Traditional use of tilianin has relied on herbal preparations such as teas, tinctures, or decoctions, with dosages varying according to practitioner experience and cultural context. By contrast, modern pharmacological research employs standardized extracts and well-defined dosing regimens to assess bioavailability, pharmacokinetics, and clinical efficacy. This evolution from empirical, experience-based dosing toward precise, evidence-driven evaluation underscores the need to bridge traditional practices with modern pharmacology in order to fully realize tilianin’s therapeutic potential.

**Fig. 1:**
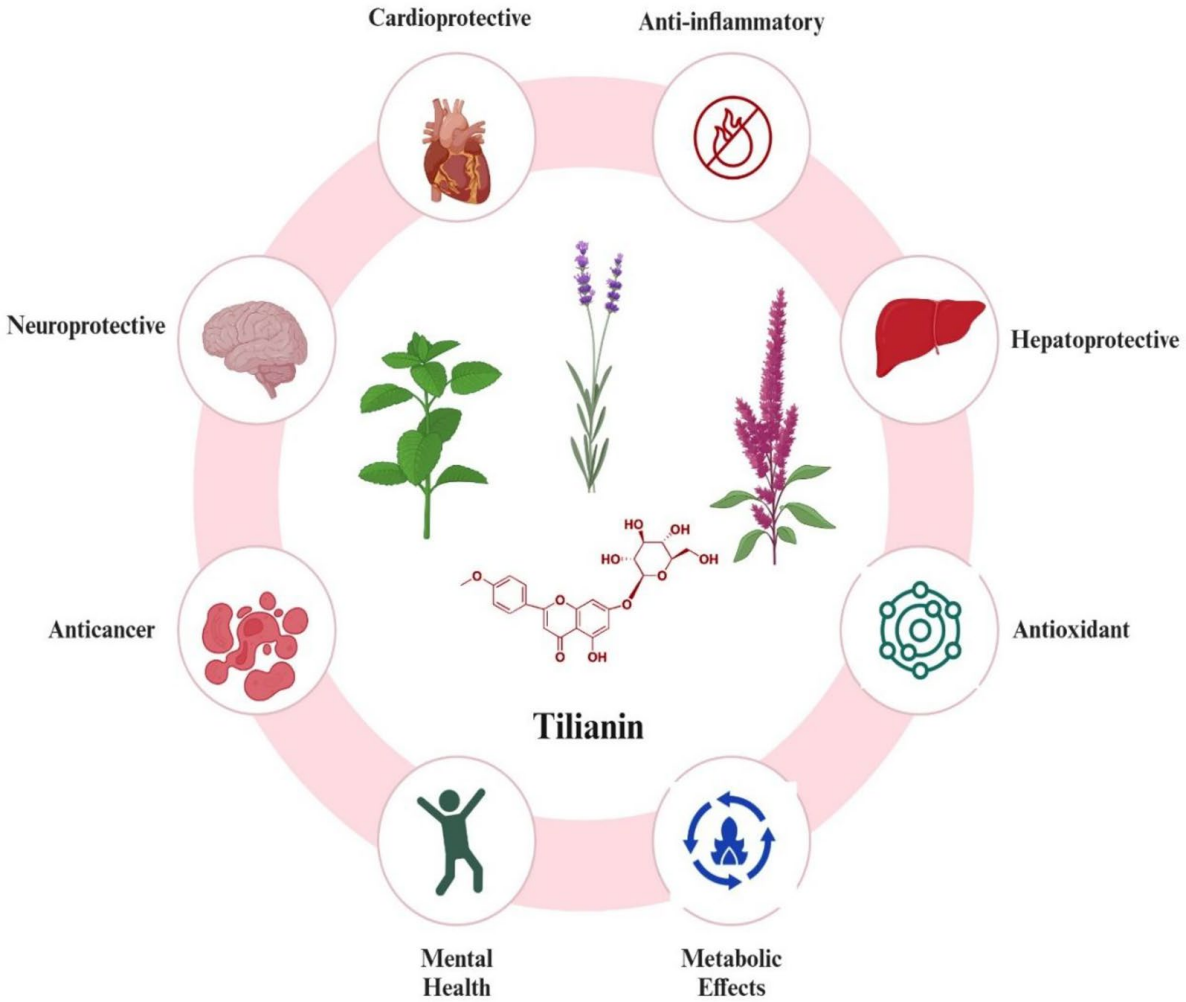
Therapeutic potential of tilianin: a multifunctional bioactive flavonoid. Chemical structure of tilianin (PubChem CID: 5321954) and its major pharmacological activities, including cardiovascular protection, neuroprotection, anticancer effects, anti-inflammatory activity, and mental health effects

Tilianin thus exemplifies the synergy between traditional remedies and modern science. Its multitargeted bioactivities—from antioxidant and anti-inflammatory effects to neuroprotection, cardioprotection, and anticancer properties—position it as a promising natural alternative for treating complex, multifactorial diseases. By advancing research and clinical validation, tilianin could become a cornerstone of next-generation integrative therapies, enhancing human health and longevity (Fig. 1).

## Pharmacokinetics and bioavailability of tilianin

The pharmacokinetics and bioavailability of tilianin are critical areas of research, given its considerable therapeutic potential. However, its poor solubility in water and extensive first-pass metabolism present substantial challenges to its effectiveness. Following oral administration, tilianin is absorbed in the gastrointestinal tract; however, its lipophilic nature limits its solubility in aqueous environments, which hampers efficient absorption. Once it enters the bloodstream, tilianin undergoes rapid metabolism in the liver via glucuronidation and sulfation. This process results in the formation of various metabolites, which are subsequently excreted through urine and feces. As a result, tilianin’s bioavailability remains low due to quick clearance and extensive first-pass metabolism [18].

To overcome these limitations, researchers are exploring advanced drug delivery strategies. One promising approach involves formulating tilianin into nanoparticles or liposomes. These nanoformulations enhance tilianin’s solubility, stability, and gastrointestinal absorption. Additionally, by bypassing some of the first-pass metabolism, they can increase its systemic concentrations. Moreover, absorption enhancers, such as piperine, which inhibit cytochrome P450 enzymes, help reduce metabolic clearance and prolong tilianin’s presence in the bloodstream [5].

Studies suggest that tilianin, whether in its native form or through advanced delivery systems, can accumulate in target tissues like the liver, kidneys, and brain, where it exerts therapeutic effects [19]. However, its low systemic exposure remains a major hurdle for clinical applications. To address this, novel formulations like solid lipid nanoparticles and self-emulsifying drug delivery systems are being developed to improve absorption and reduce clearance. These innovations aim to ensure that tilianin reaches therapeutic concentrations in tissues where its antioxidant and anti-inflammatory effects can be most beneficial [20–22] (Table 1).

**Table 1:** Pharmacokinetic characteristics, formulation, and bioavailability of tilianin

Aspect	Details for Tilianin	Refs.
Absolute oral bioavailability (%F) in mammals	Good bioavailability observed in rats	[21]
Tissue distribution	Pancreas, liver, and lung (deposited 5 h after oral administration)	[21]
PK parameters	Tmax: 1.00 h Cmax: 29.01 μg/ml T1/2: 3.33 h AUC0–t: 62.25 µg h/ml AUC0–∞: 92.47 µg h/ml Kel: 0.21 1/h Vd/F: 2,788.05 ml	[21]
Brain distribution	No specific data available	[18, 20, 21]
Brain concentrations and neuroprotection	No indication that brain concentrations of tilianin reach therapeutic levels for neuroprotection	[18, 20]
Lipid-based formulations	TCPLs were developed to enhance oral bioavailability. The optimum preparation conditions were phospholipid amount 500 mg, cholesterol amount 50 mg, and phospholipid/drug ratio of 25	[18]
Impact of lipid-based formulation on bioavailability	TCPLs resulted in 5.7 times higher Cmax and 4.6-fold higher AUC compared to tilianin solution	[18]
Metabolism and excretion	Metabolized into acacetin-7-glucuronide and acacetin-7-sulfate in mouse plasma. Not excreted through urine	[20, 21]
Permeability	Lower permeability compared to furosemide and naproxen using an everted gut model (1.43 × 10^–6^ cm/s)	[21]

*%F* absolute oral bioavailability, *Tmax* time to reach maximum concentration, *Cmax* maximum concentration, *T1/2* terminal half-life, *AUC* area under the curve, *Kel* elimination rate constant, *Vd/F* apparent volume of distribution, *PK* pharmacokinetic, *TCPL* tilianin composite phospholipid liposomes

Optimizing tilianin’s pharmacokinetics is essential for bridging the gap between preclinical research and clinical use. These advancements are crucial for maximizing its therapeutic efficacy, especially in treating chronic diseases such as cardiovascular disorders, neurodegenerative conditions, and cancer, and ensuring its potential as a powerful natural therapeutic agent.

## Phytochemical composition of tilianin

Tilianin, chemically known as acacetin-7-O-glucoside, is a flavonoid glycoside belonging to the polyphenolic class of compounds [23]. Its structure consists of acacetin covalently linked to a glucose moiety at the 7th hydroxyl position via a glycosidic bond. This modification enhances its water solubility, bioavailability, and stability compared to its aglycone form, acacetin [24]. The chromone core of tilianin, containing hydroxyl and methoxy groups, is key to its antioxidant activity. These functional groups neutralize ROS, reducing oxidative stress—a major factor in the development of diseases such as cardiovascular disorders and neurodegenerative conditions. This antioxidant activity further supports its anti-inflammatory, cardioprotective, and neuroprotective properties [25].

Given its pharmacological importance, efficient extraction methods are crucial for obtaining tilianin while preserving its bioactivity. Traditional solvent extraction using ethanol–water mixtures is widely utilized due to its efficiency and stability [4]. More advanced techniques, such as ultrasound-assisted extraction (UAE) and supercritical fluid extraction (SFE), offer improved yields and sustainability [26]. UAE uses ultrasonic waves to enhance cell wall disruption, allowing for the preservation of heat-sensitive compounds like tilianin [27]. SFE, which employs supercritical CO_2_ with ethanol as a co-solvent, ensures a high-purity extract with minimal environmental impact [26].

Following extraction, precise quantification and structural characterization are essential to confirm quality and consistency. High-performance liquid chromatography (HPLC) paired with UV or mass spectrometry provides accurate quantification and profiling of tilianin in plant extracts. Ultra-high-performance liquid chromatography further enhances resolution and speed. Nuclear magnetic resonance spectroscopy validates structural details, including glycosidic linkages, while Fourier-transform infrared spectroscopy confirms chemical composition [4].

Taken together, tilianin’s unique glycosidic structure, antioxidant-rich chromone core, and well-characterized bioactivity make it a promising therapeutic agent for cardiovascular, metabolic, and neurodegenerative disorders. Recent advances in drug delivery systems, including nanoparticles and liposomes, are being applied to optimize its therapeutic efficacy by improving targeted delivery and clinical outcomes. By integrating optimized extraction strategies, rigorous analytical validation, and innovative delivery approaches, tilianin is advancing as a candidate for future integrative healthcare solutions.

## Pharmacological properties

### Antioxidant, anti-inflammatory, and cardiovascular properties

Tilianin exerts cytoprotective effects through a combination of antioxidant, anti-inflammatory, and cardiovascular actions. Its antioxidant activity has been directly demonstrated through radical-scavenging assays, where it neutralizes hydroxyl radicals, superoxide anions, and hydrogen peroxide—reactive species implicated in cancer, neurodegeneration, and cardiovascular disorders [13]. Hydroxyl radicals, which initiate oxidative chain reactions, are directly quenched by tilianin via electron donation from its hydroxyl groups, yielding stable water molecules [28]. Tilianin has also been shown to reduce superoxide anions to molecular oxygen and hydrogen peroxide, and to scavenge hydrogen peroxide into water, thereby limiting the formation of secondary radicals [6].

**Fig. 2:**
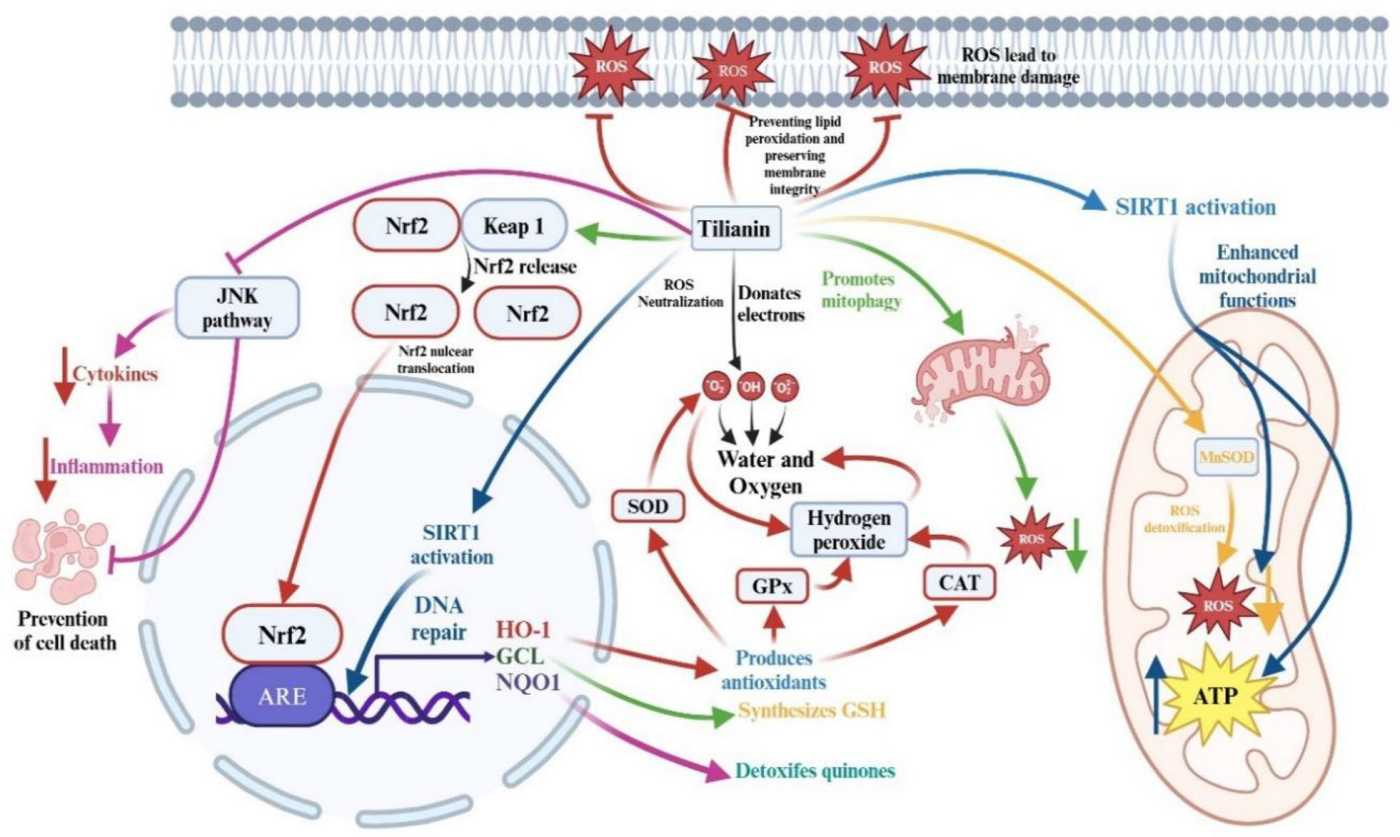
Proposed antioxidant mechanisms of tilianin. Tilianin may enhance endogenous antioxidant defenses (SOD, CAT, GPx) and activate Nrf2-driven cytoprotective genes (HO-1, GCL, NQO1), while inhibiting JNK signaling and potentially modulating SIRT1. These mechanisms are based mainly on indirect evidence from preclinical studies, and direct target validation is still needed. *ROS* reactive oxygen species, *SOD* superoxide dismutase, *CAT* Catalase, *GPx* glutathione peroxidase, *Nrf2* nuclear factor erythroid 2-related factor 2, *HO-1* heme oxygenase-1, *GCL* glutamate-cysteine ligase, *NQO1* NADPH-quinone oxidoreductase 1, *JNK* c-Jun N-terminal kinase, *SIRT1* sirtuin 1. ↑: increase, ↓: decrease, → : activate, ┬: inhibit

Beyond direct scavenging, tilianin indirectly enhances endogenous antioxidant defenses. Preclinical studies report increased activity of superoxide dismutase (SOD), catalase, and glutathione peroxidase (GPx) following treatment [29, 30]. Mechanistically, tilianin stabilizes and activates the transcription factor Nrf2, promoting its nuclear translocation and the subsequent induction of antioxidant response genes such as HO-1, glutamate-cysteine ligase, and NQO1 [31, 32]. Additional evidence suggests upstream activation of mitochondrial regulators, including SIRT1, which contributes to increased MnSOD activity, induces mitophagy, improves mitochondrial integrity, and reduces mitochondrial ROS production [14, 25, 33, 34]. These findings indicate that tilianin not only acts as a direct radical scavenger but also indirectly strengthens cellular antioxidant capacity. However, these mechanisms remain primarily supported by in vitro and animal data and require validation in human systems (Fig. 2).

Tilianin’s antioxidant actions are closely coupled to its anti-inflammatory effects. By limiting ROS-driven activation of redox-sensitive pathways, tilianin indirectly suppresses transcription factors AP-1 and NF-κB. This suppression occurs through stabilization of IκB-α, an upstream inhibitor of NF-κB, thereby reducing downstream expression of pro-inflammatory mediators including TNF-α, IL-1β, IL-6, COX-2, and iNOS [35, 36]. Tilianin has also been shown to reduce phosphorylation of MAPKs, particularly p38 MAPK, while modulating ERK1/2 and JNK activity [9, 37–39]. These upstream signaling changes result in decreased cytokine release, chemokine expression, and vascular smooth muscle proliferation.

These anti-inflammatory pathways contribute to cardiovascular protection. In endothelial cells, tilianin directly activates the PI3K/Akt pathway, which leads to phosphorylation of eNOS, increases nitric oxide (NO) bioavailability, and reduces endothelin-1 expression [8, 14, 39]. This upstream activation promotes vasodilation and preserves endothelial function. In addition, tilianin inhibits angiotensin-converting enzyme (ACE), thereby preventing downstream vascular remodeling, fibrosis, and hypertension [40]. Through these mechanisms, preclinical studies report improvements in endothelial function, blood pressure regulation, and ischemia–reperfusion injury [41]. Beyond vascular signaling, tilianin has been implicated in cardioprotection through modulation of autophagy-related proteins (Beclin-1, LC3), activation of AMPK, and regulation of circadian rhythm pathways. It also influences microRNAs such as miR-146a, which downregulates TNF-α and IL-1β signaling [14, 42]. These downstream effects collectively support mitochondrial metabolism, inhibit inflammatory cascades, and preserve cardiac tissue integrity.

Tilianin stands out among other flavonoids such as Quercetin, Luteolin, Resveratrol, and Curcumin due to its unique glycosidic structure, which enhances bioavailability, solubility, and stability. This structure allows tilianin to more effectively target therapeutic pathways, especially in diseases related to oxidative stress and inflammation. Tilianin scavenges ROS and boosts antioxidant defenses (HO-1, SOD, CAT), providing long-lasting cellular protection. It modulates inflammatory pathways such as NF-κB and MAPK, inhibiting pro-inflammatory cytokines like TNF-α and IL-6. In comparison, Apigenin and Kaempferol also target oxidative stress and inflammation, but their effects are primarily mediated through antioxidant mechanisms and NF-κB inhibition, and they lack the solubility and bioavailability enhancements that tilianin offers. This dual antioxidant and anti-inflammatory action makes tilianin particularly effective for chronic inflammation and neurodegenerative disorders [43, 44] (Table 2).

**Table 2:** Comparison of tilianin with other flavonoids across therapeutic properties

Property	Tilianin	Other flavonoids	Reference
Antioxidant and anti-inflammatory	Scavenges ROS, enhances antioxidant defenses (HO-1, SOD, CAT), modulates inflammatory pathways (NF-κB, MAPK), inhibits pro-inflammatory cytokines (TNF-α, IL-6)	Apigenin and Kaempferol target oxidative stress and inflammation mainly through antioxidant effects and NF-κB inhibition but lack solubility enhancement	[43, 44]
Neuroprotective effects	Modulates microglial activation (M1 to M2), reduces neuroinflammation and oxidative stress via PI3K/Akt	Resveratrol and Curcumin offer neuroprotection but lack Tilianin's ability to modulate microglial activation	[49, 50]
Cardioprotective properties	Protects against ischemic injury by enhancing mitochondrial function, reducing oxidative stress, and promoting autophagy	Epicatechins and Anthocyanins offer cardioprotection but lack the dual enhancement of mitochondrial function and autophagy	[45, 46]
Cancer chemoprevention	Targets apoptosis, proliferation, and angiogenesis through PI3K/Akt, MAPK, and VEGF pathways	Genistein and Quercetin mainly focus on apoptosis and cell cycle regulation, lacking a multi-target approach like Tilianin	[68, 69]
Metabolic health	Activates AMPK, PPARα, and SIRT1, improving insulin sensitivity and reducing inflammation	Rutin and Hesperidin primarily focus on antioxidant effects, without the broader metabolic regulation offered by Tilianin	[43, 75]
Hepato- and reno-protective	Targets oxidative stress and inflammation through Nrf2/ARE and NF-κB signaling, reduces liver fibrosis and prevents renal fibrosis by modulating TGF-β1	Quercetin, Luteolin, Rutin provide hepatoprotection mainly through antioxidants, while Curcumin and Silymarin are less effective at preventing fibrosis or have poor bioavailability	[68, 76, 78]

*ROS* reactive oxygen species, *HO-1* heme oxygenase-1, *SOD* superoxide dismutase, *CAT* catalase, *NF-κB* nuclear factor kappa-light-chain-enhancer of activated B cells, *MAPK* mitogen-activated protein kinase, *TNF-α* tumor necrosis factor-alpha, *IL-6* interleukin-6, *PI3K/Akt* phosphoinositide 3-kinase/protein kinase B, *VEGF* vascular endothelial growth factor, *AMPK* AMP-activated protein kinase, *PPARα* peroxisome proliferator-activated receptor alpha, *SIRT1* sirtuin 1, *TGF-β1* transforming growth factor beta 1, *Nrf2/ARE* nuclear factor erythroid 2–related factor 2/antioxidant response element

In cardioprotection, tilianin regulates mitochondrial function, reduces oxidative stress, modulates apoptosis, and promotes mitochondrial biogenesis and autophagy, which are crucial for maintaining heart tissue during ischemic events. While Epicatechins and Anthocyanins provide cardioprotection through similar mechanisms, tilianin’s simultaneous enhancement of mitochondrial function and autophagy offers a more holistic approach, ensuring better preservation and recovery of cardiac tissue [45, 46] (Table 2).

**Fig. 3:**
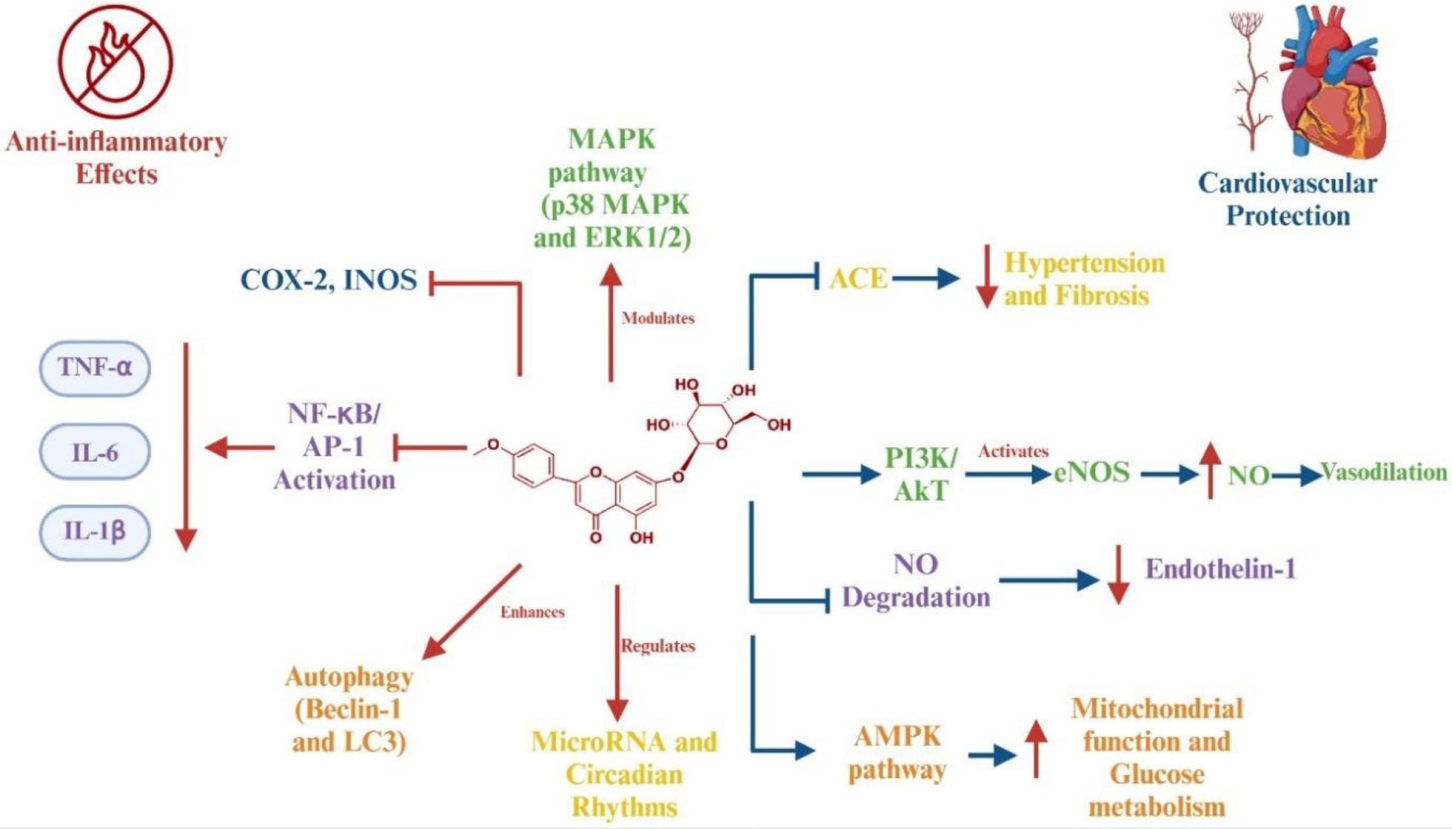
Proposed anti-inflammatory and cardiovascular protective mechanisms of tilianin. Tilianin may inhibit pro-inflammatory mediators (NF-κB, AP-1, TNF-α, IL-1β, IL-6, COX-2, iNOS) and MAPK pathways, while promoting cardioprotective signaling (eNOS, NO, PI3K/Akt, AMPK). It may also influence ACE activity and miRNA regulation. These effects are primarily supported by preclinical studies, and direct mechanistic validation is needed. *NF-κB* nuclear factor kappa B, *AP-1* activator protein 1, *TNF-α* tumor necrosis factor-alpha, *IL-1β* interleukin-1 beta, *IL-6* interleukin-6, *COX-2* cyclooxygenase-2, *iNOS* inducible nitric oxide synthase, *MAPK* mitogen-activated protein kinase, *ERK1/2* extracellular signal-regulated kinases 1/2, *LC3* microtubule-associated protein 1A/1B-Light chain 3, *eNOS* endothelial nitric oxide synthase, *PI3K/Akt* phosphoinositide 3-kinase/protein kinase B pathway, *ACE* angiotensin-converting enzyme, *AMPK* AMP-activated protein kinase. ↑: increase, ↓: decrease, →: activate, ┬: inhibit.

Taken together, tilianin exerts direct antioxidant activity, indirectly regulates redox-sensitive signaling pathways, and couples these effects with anti-inflammatory and cardiovascular protective actions. Its distinctive structural and mechanistic features confer advantages over other flavonoids, suggesting multi-target therapeutic potential against oxidative stress- and inflammation-related disorders, including hypertension, atherosclerosis, and ischemic injury. Importantly, these conclusions are based on preclinical data, and their relevance to human physiology remains to be established (Fig. 3).

### Neuroprotective properties

Tilianin demonstrates significant neuroprotective effects by directly counteracting oxidative stress, suppressing neuroinflammatory signaling, and promoting neuronal survival, making it a promising candidate for treating neurodegenerative diseases. A key mechanism proposed for these effects is its direct ROS scavenging activity, which prevents oxidative damage to neurons. Tilianin has been reported to activate the Nrf2 signaling pathway by stabilizing Nrf2 and promoting its nuclear translocation, thereby upregulating antioxidant enzymes such as HO-1, SOD, GPx, and catalase. These enzymes indirectly preserve mitochondrial integrity, sustain ATP production, and inhibit the release of pro-apoptotic factors such as cytochrome c, thereby supporting neuronal function and survival [9, 13, 47].

In addition to its antioxidant properties, tilianin directly inhibits NF-κB pathway activation, reducing the transcription of pro-inflammatory cytokines including TNF-α, IL-1β, and IL-6 [11]. Tilianin has also been shown to shift microglial polarization from a pro-inflammatory (M1) state to an anti-inflammatory (M2) phenotype, supporting tissue repair and reducing the release of neurotoxic mediators such as ROS and reactive nitrogen species [48]. This ability to modulate microglial activation is a distinctive feature of tilianin, not shared by other well-known neuroprotective flavonoids such as Resveratrol or Curcumin, making it a more comprehensive agent for targeting both neuroinflammation and oxidative stress in neurodegenerative diseases [49, 50] (Table 2).

Furthermore, tilianin may modulate monoamine oxidase (MAO) activity, although studies suggest that tilianin itself exhibits limited MAO inhibition compared to acacetin derivatives; modifications at the sugar moiety can significantly enhance inhibitory potency (IC₅₀ in the low micromolar range) [51]. Tilianin has been proposed to activate the PI3K/Akt pathway, which inhibits pro-apoptotic proteins such as Bax and upregulates anti-apoptotic proteins such as Bcl-2, thereby promoting neuronal survival. It may also attenuate excitotoxicity by enhancing GABAergic neurotransmission and limiting intracellular calcium overload, a critical trigger of neuronal death [13, 52]. Recent studies further support tilianin’s antioxidant and anti-inflammatory actions in vivo: extracts from *Agastache* species rich in tilianin reduced pro-oxidant biomarkers (TOS, OSI, MDA, NO) and enhanced antioxidant markers (TAC, SH groups), while also providing cardioprotective effects [47].

By inhibiting toll-like receptor 4 (TLR4) signaling, tilianin reduces microglial activation and the production of pro-inflammatory cytokines, contributing to its anti-inflammatory profile [38, 53, 54]. Additionally, direct inhibition of glycogen synthase kinase-3β (GSK-3β) has been observed, which reduces tau hyperphosphorylation and neurofibrillary tangle formation in models of Alzheimer’s disease [13]. Tilianin may also support cognitive function by upregulating microRNAs such as miR-132, which enhances synaptic plasticity, and miR-146a, which suppresses inflammatory signaling [55].

Tilianin’s neuroprotective actions may also involve autophagy. It has been reported to upregulate autophagy-related proteins such as LC3 and Beclin-1, thereby promoting the clearance of damaged proteins and organelles. Furthermore, tilianin has been suggested to strengthen blood–brain barrier (BBB) integrity by upregulating tight junction proteins, thereby limiting neuroinflammation [56]. It may also influence the PI3K/Akt/mTOR pathway, which supports neuronal survival, synaptic plasticity, and memory formation [12].

**Fig. 4:**
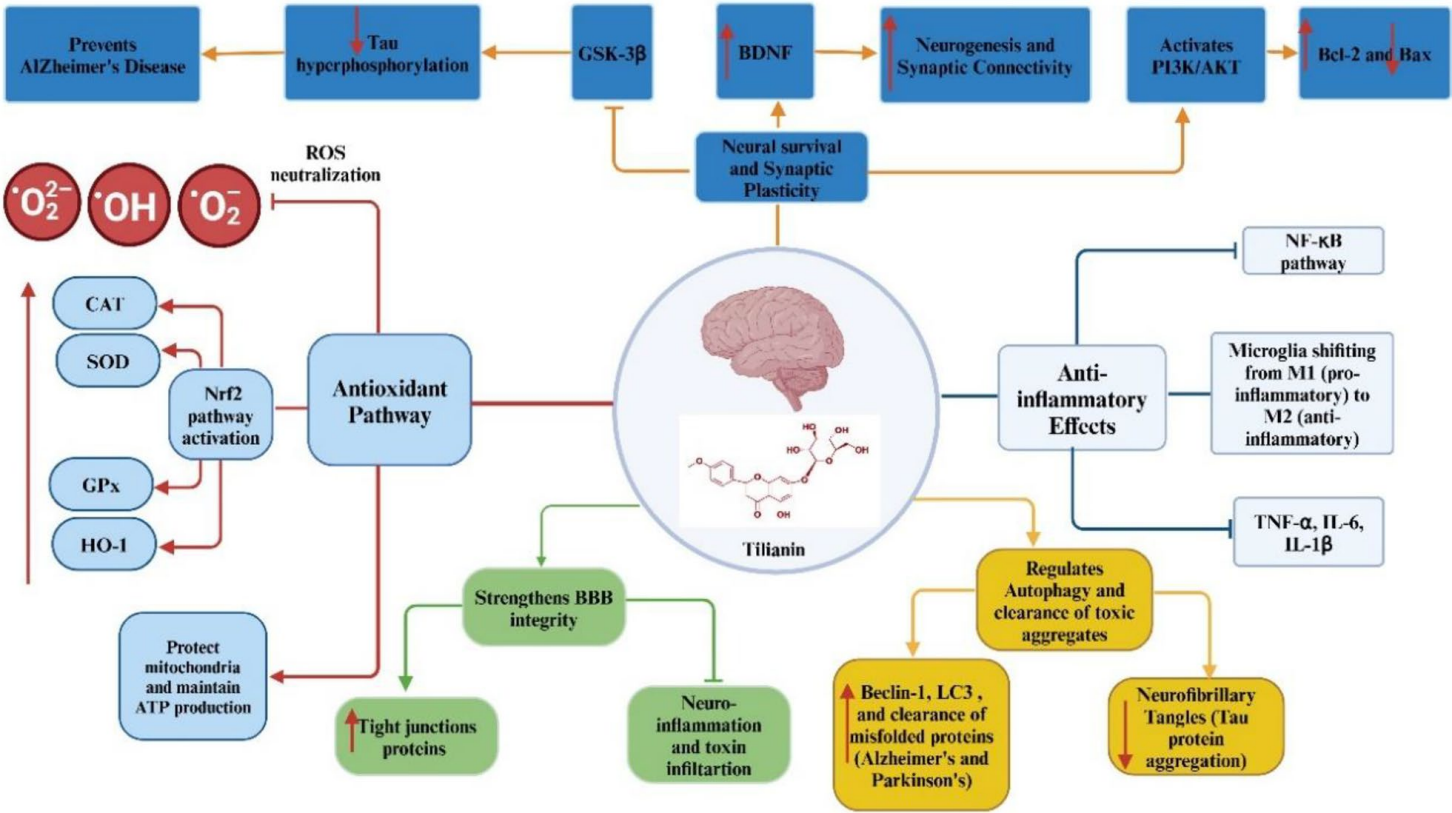
Proposed neuroprotective actions of tilianin: antioxidant, anti-inflammatory, and cognitive support. Tilianin may reduce oxidative stress (ROS, Nrf2/HO-1, SOD, GPx, CAT), suppress neuroinflammation (NF-κB, TNF-α, IL-1β, IL-6), and modulate survival and plasticity pathways (PI3K/Akt, Bax/Bcl-2, BDNF, GSK-3β). Additional roles include preserving BBB integrity and regulating autophagy (LC3). These mechanisms are primarily based on preclinical findings, and direct experimental validation remains necessary. *ROS* reactive oxygen species, *Nrf2* nuclear factor erythroid 2-related factor 2, *HO-1* heme oxygenase-1, *SOD* superoxide dismutase, *GPx* glutathione peroxidase, *CAT* catalase, *NF-κB* nuclear factor kappa B, *TNF-α* tumor necrosis factor-alpha, *IL-1β* interleukin-1 beta, *IL-6* interleukin-6, *PI3K/Akt* phosphoinositide 3-kinase/protein kinase B pathway, *Bax* Bcl-2 associated X protein, *Bcl-2* B-cell lymphoma, *BDNF* brain-derived neurotrophic factor, *GSK-3β* glycogen synthase kinase-3 beta, *BBB* blood–brain barrier, *LC3* microtubule-associated protein 1A/1B-light chain 3. ↑: increase, ↓: decrease, →: activate, ┬: inhibit.

Taken together, tilianin integrates direct antioxidant defense, indirect enhancement of endogenous neuroprotective pathways, suppression of inflammatory cascades, and regulation of neuronal survival mechanisms. Its measured in vitro antioxidant activity (DPPH IC₅₀ ≈ 66–69 μg/mL) and in vivo efficacy underscore its therapeutic potential for neurodegenerative diseases, ischemic injury, and other neurological disorders [47]. Importantly, tilianin’s dual capacity to reduce oxidative stress and neuroinflammation—alongside its unique ability to shift microglial activation from M1 to M2—confers superior neuroprotection compared with flavonoids like Resveratrol or Curcumin [49, 50] (Table 2). As these findings are primarily derived from preclinical studies, they remain provisional and require direct experimental validation to confirm molecular targets and clinical relevance (Fig. 4).

### Cancer chemoprevention

Tilianin has been reported to exhibit chemopreventive potential by directly targeting multiple molecular pathways involved in cancer initiation, progression, and metastasis. One proposed mechanism is the activation of the Nrf2 signaling cascade, which induces transcription of antioxidant enzymes such as HO-1, SOD, and GPx. This activity reduces oxidative stress and limits DNA damage, thereby lowering the likelihood of carcinogenesis. Concurrently, tilianin inhibits NF-κB activation, leading to reduced production of pro-inflammatory cytokines including TNF-α, IL-6, and IL-1β, which otherwise promote tumor proliferation and survival [57].

Tilianin induces G0/G1 cell cycle arrest through downregulation of cyclin D1, CDK4, and CDK2, and triggers apoptosis by shifting the Bcl-2/Bax ratio toward pro-apoptotic signaling [58, 59]. It may also enhance autophagy-dependent cell death in malignant cells by increasing Beclin-1 and LC3 expression, while sparing normal cell viability [60]. IC₅₀ values reported in thyroid carcinoma and non-small cell lung cancer (NSCLC) models range from 10 to 50 μM, indicating dose-dependent cytotoxicity in tumor cells [61, 62].

A notable anti-cancer effect of tilianin is its reported inhibition of angiogenesis. By suppressing VEGF transcription and VEGFR signaling, tilianin disrupts tumor-associated blood vessel formation, thereby restricting nutrient supply and metastatic potential [57, 58]. Tilianin also alters microRNA expression, including upregulation of tumor-suppressor miRNAs that suppress oncogenes and inflammatory mediators, contributing to reduced tumor progression [63, 64].

Epigenetic mechanisms have been implicated in tilianin’s activity. Tilianin reportedly increases histone acetylation and promotes DNA demethylation, reactivating tumor-suppressor genes. Additionally, it blocks JAK/STAT signaling by inhibiting STAT3 phosphorylation, a critical driver of cancer cell proliferation, immune evasion, and stemness. Consistent with this, tilianin downregulates matrix metalloproteinases (MMP-2 and MMP-9), thereby reducing extracellular matrix degradation and tumor invasion [65, 66].

Recent studies suggest that tilianin suppresses PI3K/Akt/mTOR signaling, reducing Akt and mTOR phosphorylation, which disrupts cancer cell proliferation and promotes autophagy under metabolic stress [58, 63]. It also inhibits fibroblast activation and epithelial-to-mesenchymal transition (EMT) by downregulating TGF-β and SMAD signaling, attenuating tumor cell migration and invasion [58, 67]. Tilianin further inhibits tumor growth by modulating MAPK pathways, demonstrating a dual impact on proliferation and survival [68, 69] (Table 2). Unlike Genistein and Quercetin, which primarily focus on apoptosis and cell cycle regulation, tilianin simultaneously targets multiple cancer-related pathways, making it a more comprehensive agent for cancer prevention and treatment [68, 69] (Table 2).

Tilianin’s pro-apoptotic activity may involve the extrinsic pathway, as it activates FasR and TRAIL-R death receptors, increasing susceptibility of tumor cells to programmed cell death [58, 70]. In parallel, it influences the DNA damage response by promoting repair in normal cells while enhancing checkpoint activation in cancer cells, impairing malignant progression [71, 72]. Furthermore, tilianin inhibits oncogenic Notch and Wnt/β-catenin signaling, suppressing Notch1 and β-catenin activity, which reduces tumor proliferation, cancer stem cell renewal, and metastatic potential. It also inhibits histone deacetylases, enhancing tumor-suppressor gene transcription and strengthening anti-tumor immune responses [25, 65].

**Fig. 5:**
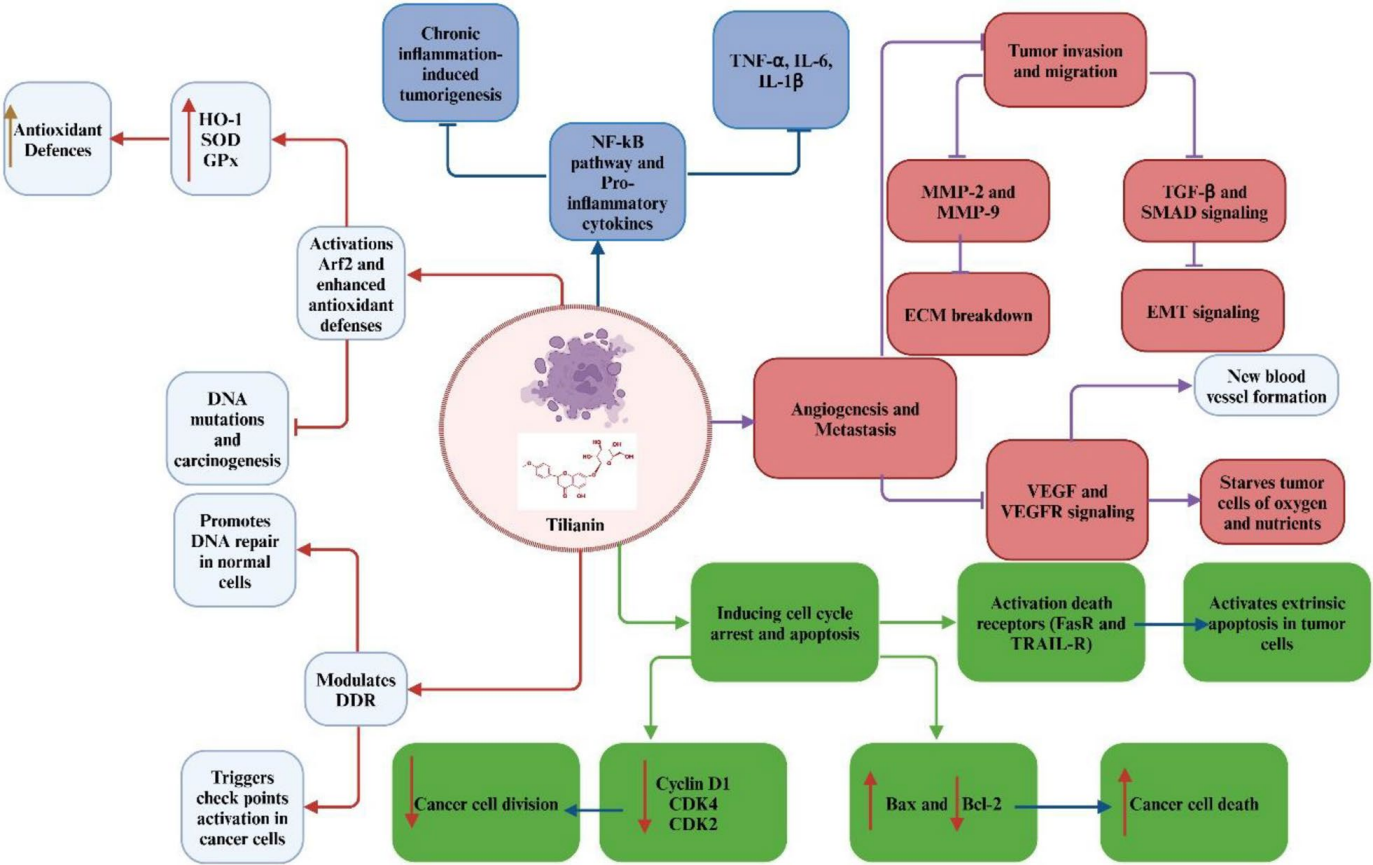
Proposed chemopreventive actions of tilianin. Tilianin may exert anticancer effects by activating antioxidant pathways (Nrf2/HO-1, SOD, GPx), suppressing inflammation (NF-κB, TNF-α, IL-6), inhibiting angiogenesis (VEGF/VEGFR), and regulating tumor growth and metastasis via PI3K/Akt/mTOR, MMP-2, MMP-9, TGF-β, and EMT processes. These mechanisms are largely based on in vitro and animal studies, and direct molecular validation is still required. *Nrf2* nuclear factor erythroid 2-related factor 2, *HO-1* heme oxygenase-1, *SOD* superoxide dismutase, *GPx* glutathione peroxidase, *NF-κB* nuclear factor kappa B, *TNF-α* tumor necrosis factor-alpha, *IL-6* interleukin-6, *VEGF* vascular endothelial growth factor, *VEGFR* vascular endothelial growth factor receptor, *PI3K/Akt/mTOR* phosphoinositide 3-kinase/protein kinase B/mechanistic target of rapamycin, *MMP-2* matrix metalloproteinase-2, *MMP-9* matrix metalloproteinase-9, *TGF-β* transforming growth factor-beta, *EMT* epithelial–mesenchymal transition. ↑: increase, ↓: decrease, → : activate, ┬: inhibit

In conclusion, tilianin’s chemopreventive actions involve direct modulation of oxidative stress, inflammatory signaling, cell cycle progression, apoptosis, angiogenesis, epigenetic programs, and oncogenic pathways such as PI3K/Akt/mTOR, MAPK, JAK/STAT, and Wnt/β-catenin. Its demonstrated in vitro cytotoxicity, IC₅₀ data, and multi-pathway mechanistic profile underscore its potential as a chemopreventive and adjuvant therapeutic agent. However, these mechanisms are largely derived from preclinical and indirect evidence, and further validation is required to confirm its molecular targets, improve bioavailability, and establish clinical efficacy and safety (Fig. 5).

### Therapeutic potential of tilianin in metabolic disorders

Tilianin has demonstrated significant potential as a therapeutic agent for metabolic disorders by directly activating AMP-activated protein kinase (AMPK), which stimulates glucose uptake in skeletal muscle and adipose tissue and enhances fatty acid oxidation [35]. This AMPK activation inhibits acetyl-CoA carboxylase, thereby suppressing lipid accumulation, improving lipid metabolism, and increasing insulin sensitivity while reducing blood glucose levels [73].

Tilianin also modulates nuclear receptors, particularly peroxisome proliferator-activated receptors (PPARs) such as PPARα and PPARγ. Activation of PPARα increases fatty acid oxidation in the liver and muscle, reducing ectopic fat deposition and improving insulin sensitivity [15, 74]. Activation of PPARγ in adipose tissue promotes lipid storage and utilization, contributing to metabolic balance [64]. Additionally, tilianin activates SIRT1, which induces mitochondrial biogenesis, oxidative phosphorylation, and energy production. SIRT1 also enhances insulin sensitivity, stimulates lipid oxidation, and suppresses lipogenesis, offering benefits for managing obesity and type 2 diabetes [66].

Through the gut-liver axis, tilianin alters the gut microbiome, increasing the production of secondary bile acids that activate the farnesoid X receptor (FXR) in the liver. This FXR activation reduces hepatic triglyceride accumulation and improves lipid metabolism, providing protection against MAFLD. Tilianin also stimulates brown adipose tissue (BAT) activity by upregulating thermogenic genes such as uncoupling protein 1, thereby increasing energy expenditure, combating obesity, and improving fat metabolism [67]. In addition to these effects, tilianin inhibits GSK-3β, which promotes glycogen synthesis in the liver and supports glucose homeostasis. Its anti-inflammatory and antioxidant properties further improve insulin signaling by suppressing TNF-α and IL-6 expression, thereby attenuating insulin resistance and metabolic dysfunction [42, 67].

Tilianin’s regulation of AMPK, PPARα, PPARγ, SIRT1, gut microbiota, BAT activity, glycogen synthesis, and inflammatory signaling underscores its potential as a multi-target therapeutic agent for metabolic disorders such as obesity, insulin resistance, type 2 diabetes, and MAFLD. Unlike Rutin and Hesperidin, which mainly exert antioxidant effects, tilianin combines modulation of metabolic regulators with antioxidant activity, offering a more comprehensive approach to managing metabolic dysfunction [43, 75] (Table 2).

### Hepato- and reno-protective properties

Tilianin demonstrates significant protective effects in both liver and kidney tissues by counteracting oxidative stress, inflammation, and fibrosis. In hepatic models, it activates the Nrf2/ARE pathway, upregulating antioxidant enzymes such as HO-1, SOD, and CAT. This activity reduces lipid peroxidation, preserves mitochondrial function, and attenuates injury in conditions including MAFLD, hepatitis, and fibrosis. Tilianin also inhibits NF-κB signaling, suppressing pro-inflammatory cytokines such as TNF-α and IL-6, while blocking hepatic stellate cell activation to limit collagen deposition and cirrhosis [70, 72]. Compared with other flavonoids, tilianin offers superior hepatoprotection. Quercetin, Luteolin, and Rutin mainly exert antioxidant effects and are less effective at preventing liver fibrosis. Curcumin provides anti-inflammatory benefits but suffers from low bioavailability, limiting its therapeutic potential, while Silymarin lacks the dual action on oxidative stress and fibrosis that tilianin provides [76].

In renal tissues, tilianin enhances antioxidant defenses (GPx, SOD, CAT), suppresses NF-κB–mediated inflammation, and modulates TGF-β1 signaling to prevent fibrosis. These effects preserve renal tubular integrity, glomerular filtration, and blood flow, protecting against diabetic nephropathy, chronic kidney disease, and acute kidney injury [77]. Compared to other compounds, Quercetin and Rutin primarily offer antioxidant protection but are less effective in regulating fibrosis and TGF-β1 signaling. Luteolin and Curcumin reduce inflammation to some extent but are limited by lower bioavailability, while Catechins provide antioxidant protection without significant impact on fibrosis or overall kidney function [68, 78, 79] (Table 2).

Across both organs, tilianin modulates the AMPK/mTOR pathway to promote autophagy and activates SIRT1, enhancing mitochondrial biogenesis, oxidative phosphorylation, and cellular stress resistance [14, 73]. These integrated mechanisms decrease hepatic steatosis, fibrosis, and insulin resistance while strengthening renal resilience to metabolic disturbances. Overall, tilianin surpasses other flavonoids in both hepatoprotective and renoprotective properties due to its ability to simultaneously target oxidative stress, inflammation, and fibrosis. Its enhanced bioavailability and multi-pathway modulation (Nrf2, NF-κB, TGF-β1) position tilianin as a promising agent for managing chronic liver and kidney diseases.

## Research gaps and future prospects

### Need for clinical translation

While tilianin demonstrates remarkable therapeutic potential, several research gaps must be addressed before its pharmacological benefits can be fully realized in clinical settings. Although preclinical studies have provided valuable insights, the majority of evidence comes from in vitro and animal models. To bridge this gap, well-designed clinical trials are essential to confirm tilianin’s safety, bioavailability, and efficacy in humans.

### Pharmacokinetic limitations

One of the most pressing challenges is tilianin’s pharmacokinetics. Like many flavonoids, it suffers from poor absorption and rapid metabolism, resulting in low bioavailability. Future research should prioritize optimizing its formulation using advanced drug delivery systems, such as liposomes and nanoparticles. These strategies could enhance its solubility, stability, and therapeutic efficacy, paving the way for more effective clinical applications.

### Mechanistic uncertainty and synergistic potential

While tilianin’s pharmacological effects are well-documented, its precise molecular mechanisms remain underexplored. Further studies are needed to elucidate the specific signaling pathways and receptors it targets, particularly in complex diseases such as cancer and neurodegenerative disorders. Additionally, there is significant potential for tilianin to act synergistically with other treatments. Investigating such combinations could maximize its therapeutic impact and open new avenues for clinical application.

### Clinical evidence gaps

To date, no published clinical trials or substantial human studies on tilianin are available. Current knowledge is derived almost entirely from in vitro and animal models. While these studies consistently demonstrate antioxidant, anti-inflammatory, neuroprotective, and anticancer activities, confirmation in humans is lacking. Moreover, no studies have directly compared tilianin’s efficacy with clinically approved agents, such as donepezil for neurodegenerative disorders, sorafenib for cancer, or standard antioxidants. This represents a critical gap, highlighting the need for future investigations to quantify tilianin’s therapeutic potency relative to established treatments. Future research must prioritize clinical investigations to determine tilianin’s pharmacokinetics, safety, tolerability, and efficacy, including whether therapeutic concentrations are achievable in target tissues such as the brain. Bridging this preclinical-to-clinical gap is essential before tilianin’s therapeutic promise can be realized in practice.

### Safety and toxicology

Tilianin has demonstrated a favorable safety profile in multiple preclinical studies, supporting its potential for therapeutic use. In mice, administration of tilianin up to 1000 mg/kg produced no observable toxicity, while higher doses caused only temporary lethargy lasting approximately five hours, after which animals fully recovered [41]. Additional toxicological assessments, including QSAR-based predictions in rats, estimated oral LD50 values ranging from 1060.12 mg/kg to 2622.95 mg/kg, depending on the method used [80]. In vitro studies using H9c2 cells exposed to oxygen–glucose deprivation/reperfusion (OGD/R) revealed no significant cytotoxicity compared with controls, further confirming its low toxicity [81]. Acute and sub-acute studies in Imprinting Control Region (ICR) mice and spontaneously hypertensive rats (SHR) reported an LD50 of 6624 mg/kg and an ED50 of 53.51 mg/kg, with no effects on body weight, liver enzymes, cholesterol, glucose, insulin, or the function of vital organs, including the kidney, heart, liver, and lungs [41]. Collectively, these findings indicate that tilianin is generally well tolerated even at high doses and with long-term administration.

Despite this encouraging evidence, comprehensive toxicological data for tilianin remain limited. To date, no published studies have reported its maximum tolerated dose (MTD), organ-specific toxicity evaluated through histopathology, particularly in the liver and kidneys, or potential drug–drug interactions, including modulation of cytochrome P450 (CYP450) enzymes or pregnane X receptor (PXR) activation. A focused literature search using combinations of the terms “tilianin” with CYP450, CYP, PXR, or MTD returned no relevant results, confirming the absence of these critical data. While the existing studies provide preliminary reassurance of safety, their limitations—including short study durations, lack of systematic histopathological assessment, and absence of interaction studies—preclude a complete characterization of tilianin’s toxicological profile.

Future research must address these gaps to enable clinical translation. Investigations are needed to establish safe dosage ranges and determine the MTD in relevant animal models, as well as to evaluate organ-specific toxicity with a focus on hepatic and renal tissues, including detailed histopathological analyses. Long-term subchronic and chronic exposure studies will be critical to clarify the effects of prolonged administration, and systematic studies of CYP450- or PXR-mediated drug interactions are required to define compatibility with other therapies. Addressing these knowledge gaps will be essential to fully define the safety profile of tilianin and ensure its safe application in clinical settings. Table 3 summarizes the currently available preclinical toxicological data, highlighting its generally non-toxic profile while underscoring the areas requiring further investigation.

**Table 3:** Toxicity profile of tilianin in experimental models

Study/model	Dose range	Toxicity results	Key findings	Refs.
Mice	Up to 1000 mg/kg	No toxicity observed at 1000 mg/kg; higher doses caused temporary lethargy for 5 h, followed by recovery	Tilianin is non-toxic at doses up to 1000 mg/kg	[41]
Rodents	LD50: 6624 mg/kg	No adverse effects on body weight, liver enzymes, cholesterol, glucose, insulin, or organ functions (kidney, heart, liver, lungs)	LD50 of 6624 mg/kg and ED50 of 53.51 mg/kg; no toxic effects with sub-acute administration	[41]
QSAR toxicity predictions	–	Predicted oral LD50 values for rats: 1060.12 mg/kg, 755.40 mg/kg, 2622.95 mg/kg (based on different estimation methods)	Further supports tilianin’s safety with predictions indicating safe levels at significant doses	[80]
H9c2 cells	–	No significant difference in cell viability between control and treated groups in OGD/R-induced damage	Further confirms tilianin’s non-toxic profile in cell-based assays	[81]

*LD50* median lethal dose, *ED50* median effective dose, *QSAR* quantitative structure–activity relationship, *OGD/R* oxygen–glucose deprivation/reperfusion, *ICR* imprinting control region, *SHR* spontaneously hypertensive rats

### Broader applications and ethnopharmacology

Tilianin holds great promise in addressing chronic diseases such as cancer, cardiovascular disorders, diabetes, and neurodegenerative conditions. Exploring its use as a preventive treatment for high-burden chronic diseases could significantly broaden its therapeutic scope and public health impact. Its ethnopharmacological significance also remains underexplored. Investigating its cultural and regional uses could uncover new therapeutic applications, adding to its versatility.

### Regulatory and commercialization challenges

Finally, regulatory challenges must be addressed to bring tilianin to market as a commercially viable drug. Research should focus on clarifying regulatory requirements, standardizing extracts, and ensuring consistent product quality. These efforts will be critical for tilianin’s approval and successful commercialization, ultimately making it accessible to patients worldwide.

## Summary

Tilianin, a flavonoid glycoside from medicinal plants, has shown significant pharmacological potential due to its antioxidant, anti-inflammatory, neuroprotective, cardioprotective, anticancer, hepatoprotective, renoprotective, and metabolic-regulating effects. Its unique molecular structure enhances bioavailability, stability, and solubility, making it effective in managing chronic diseases such as cardiovascular disorders, neurodegenerative diseases, diabetes, and cancer.

Tilianin’s antioxidant properties protect cells from oxidative damage by scavenging ROS and upregulating defenses like HO-1. It also modulates inflammatory pathways such as NF-κB and AP-1, offering potential for managing chronic inflammation. Additionally, tilianin protects neurons from oxidative stress and neuroinflammation, indicating promise in treating neurodegenerative diseases like Alzheimer’s and Parkinson’s. It also mitigates ischemic injury and regulates mitochondrial function, providing cardiovascular benefits.

In cancer therapy, tilianin induces apoptosis and inhibits cancer cell proliferation through key pathways like PI3K/Akt and MAPK. It also improves glucose and lipid metabolism, enhances insulin sensitivity, and regulates adiponectin levels, indicating potential for treating diabetes and metabolic syndrome. Additionally, tilianin protects the liver and kidneys, demonstrating hepatoprotective and renoprotective effects.

Despite its therapeutic promise, tilianin faces challenges such as poor bioavailability and a lack of clinical trials. Future research should focus on improving pharmacokinetics and conducting comprehensive clinical studies to optimize its use in therapeutic practice.

## Conclusion

Tilianin stands out as a promising natural therapeutic agent with wide-ranging applications for managing oxidative stress, inflammation, neurodegenerative diseases, cancer, cardiovascular conditions, metabolic disorders, and wound healing. Its diverse pharmacological activities, including antioxidant, anti-inflammatory, neuroprotective, cardioprotective, anticancer, hepatoprotective, and metabolic-regulatory effects, make it a strong candidate for treating multifactorial diseases, such as neurodegenerative disorders, cancer, and chronic liver and kidney diseases.

One of tilianin's key strengths is its ability to modulate critical biological pathways, including oxidative stress, inflammation, mitochondrial function, and apoptosis, positioning it not only as a treatment for existing conditions but also as a potential preventive agent for chronic and aging-related diseases. This unique pharmacological profile makes tilianin a promising natural alternative in integrative healthcare.

However, to fully realize its clinical potential, several challenges must be addressed, such as poor bioavailability, lack of standardized extraction methods, and limited optimization of its pharmacokinetics. Additionally, the absence of well-designed clinical trials and comprehensive toxicity studies limits its practical application. Future research should focus on enhancing pharmacokinetics, exploring advanced drug delivery systems, and conducting rigorous clinical trials. Moreover, tilianin’s potential in combination therapies could offer a synergistic approach to treating complex diseases, improving efficacy, reducing side effects, and overcoming drug resistance. Its traditional medicinal significance also invites exploration of regional applications and new therapeutic uses.

In conclusion, despite existing challenges, tilianin’s remarkable pharmacological properties make it a promising candidate for future integrative health solutions. Continued research and clinical development could position it as a cornerstone of plant-based therapies, advancing sustainable and effective treatments for complex diseases.

## Data Availability

No datasets were generated or analysed during the current study.

## Abbreviations

Akt: Protein kinase B
AMPK: AMP-activated protein kinase
AP-1: Activator protein 1
Bax: Bcl-2-associated X protein
Bcl-2: B-cell lymphoma 2
CKD: Chronic kidney disease
COX-2: Cyclooxygenase-2
GABA: Gamma-aminobutyric acid
GPx: Glutathione peroxidase
GSK-3β: Glycogen synthase kinase-3 beta
HO-1: Heme oxygenase-1
IL-6: Interleukin 6
iNOS: Inducible nitric oxide synthase
JNK: C-Jun N-terminal kinase
MAPK: Mitogen-activated protein kinase
MMP: Matrix metalloproteinases
mTOR: Mechanistic target of rapamycin
MAFLD: Metabolic dysfunction-associated non-alcoholic fatty liver disease
NF-κB: Nuclear factor kappa B
NO: Nitric oxide
Nrf2: Nuclear factor erythroid 2-related factor 2
PI3K/Akt: Phosphoinositide 3-kinase/protein kinase B
PPAR-α: Peroxisome proliferator-activated receptor alpha
ROS: Reactive oxygen species
SIRT1: Sirtuin 1
SOD: Superoxide dismutase
SREBP-1c: Sterol regulatory element-binding protein 1c
TGF-β: Transforming growth factor beta
TLR4: Toll-like receptor 4
TNF-α: Tumor necrosis factor alpha
VEGF: Vascular endothelial growth factor
